# SourceApp: A Novel Metagenomic Source Tracking Tool that can Distinguish between Fecal Microbiomes Using Genome-To-Source Associations Benchmarked Against Mixed Input Spike-In Mesocosms

**DOI:** 10.1021/acs.est.5c03603

**Published:** 2025-05-06

**Authors:** Blake G. Lindner, Katherine E. Graham, Jacob R. Phaneuf, Janet K. Hatt, Konstantinos T. Konstantinidis

**Affiliations:** † School of Civil and Environmental Engineering, 1372 Georgia Institute of Technology, Atlanta 30332, Georgia, United States; ‡ School of Biological Sciences, Georgia Institute of Technology, Atlanta 30332, Georgia, United States

**Keywords:** shotgun metagenomics, microbial source tracking, bioinformatics, fecal pollution, forensics, comparative genomics

## Abstract

Methodologies utilizing metagenomics are attractive to fecal source tracking (FST) aims for assessing the presence and proportions of various fecal inputs simultaneously. Yet, compared to established culture- or PCR-based techniques, metagenomic approaches for these purposes are rarely benchmarked or contextualized for practice. We performed shotgun sequencing experiments (*n* = 35) of mesocosms constructed from the water of a well-studied recreational and drinking water reservoir spiked with various fecal (n = 6 animal sources, 3 wastewater sources, and 1 septage source) and synthetic microbiome spike-ins (*n* = 1) introduced at predetermined cell concentrations to simulate fecal pollution events of known composition. We built source-associated genome databases using publicly available reference genomes and metagenome assembled genomes (MAGs) recovered from short- and long-read sequencing of the fecal spike-ins, and then created an associated bioinformatic tool, called SourceApp, for inferring source attribution and apportionment by mapping the metagenomic data to these genome databases. SourceApp’s performance varied substantially by source, with cows being underestimated due to under sampling of cow fecal microbiomes. Parameter tuning revealed sensitivity and specificity near 0.90 overall, which exceeded all alternative tools. SourceApp can assist researchers with analyzing and interpreting shotgun sequencing data and developing standard operating procedures on the frontiers of metagenomic FST.

## Graphical abstract

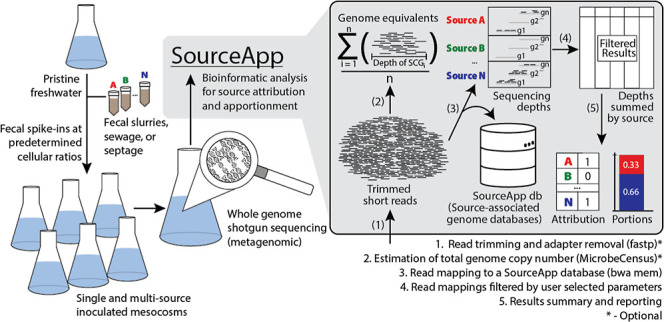

## 1 Introduction

Modern approaches for tracking fecal pollution in environmental waters constitute a discipline referred to as fecal source tracking (FST). FST methods are essential sanitation tools since fecal pollution can harbor human pathogens and harm aquatic ecosystems by introducing excess nutrients among other contaminants.^1^ Like other procedures in environmental monitoring, FST approaches aim to characterize the source and load of pollutants, e.g., fecally associated microbial populations. Two of the main objectives of FST are source attribution and source apportionment. Source attribution aims to indicate the presence or absence of contaminating source(s) while source apportionment seeks to quantify the contributions of each contaminating source individually or collectively (e.g., as ratios).^2,3^ Data streams generated from robust FST methodologies for attributing and apportioning fecal contamination in environmental waters, especially those waters used for recreation and drinking, can drive efficient engineering interventions, monitoring efforts, and decision making in support of public health.^4–6^

Culturing fecal indicator bacteria (FIB) is the primary methodology for tracking fecal contamination in the environment and is used for determining a waterbody’s regulatory compliance.^7,8^ Yet, FIB-based approaches are limited by poor source specificity and varying degrees of both persistence and prevalence in the environment.^9–11^ These challenges thwart clear insights into source attribution and apportionment, complicating the practitioner’s ability to evaluate remedial activities and make public health decisions. In response to these limitations, modern FST approaches attempt to accurately attribute the source(s) of fecal contamination in the environment based on source-associated genetic markers (e.g., sewage-associated HF183 marker). The performance and capabilities of these methods can vary substantially across sites depending on the prevalence and specificity of the genetic markers used.^12–15^ Yet, evaluating the use of these methodologies for source apportionment has been less frequent as it usually relies on a mass balance of inputs from different sources – difficult to define for most ecosystems—and incorporation of multiple genetic targets each with high source-specificities, prevalence, and equal (or known) copy numbers.^3^ Thus, further evaluation of the efficacy of emerging techniques for source apportionment is crucial for advancing FST.^16^

Our growing understanding of the structure of prokaryotic diversity, including the existence of species therein, has been driven by massive sequencing efforts across the world.^17–20^ These efforts have produced large quantities of reference genomes—some of which seem to have relatively stable host/habitat ranges, which bodes well for the tractability of microbial pollution in the environment.^21,22^ Thus, the use of whole genome shotgun sequencing of environmental samples, i.e., metagenomics, in FST has been sought after. Some of the benefits which could be provided by robust metagenomic FST methodologies include the ability to harness thousands of source-associated genomes simultaneously and the ability to apportion resulting population-level signals to potential sources. The inherent compositionality of metagenomic data sets has been extensively explored for use in broader ecologically focused frameworks such as efforts to deduce the origin of populations within microbial communities.^23–25^ Yet, specific FST-oriented approaches are largely limited to 16S rRNA gene metabarcoding.^26,27^

Currently, most of the sequencing-based approaches mentioned above have not been developed for distinguishing between specific fecal sources but instead between broad “enteric” or “gut” source categories occurring among other microbiomes (e.g., soil, freshwater, marine, etc.). Additionally, existing approaches often rely on extensive sampling of possible sources to serve as reference samples which can be impractical in many FST scenarios.^24,28,29^ These approaches have also not been developed against testing data sets with clear ground truths despite benchmarking efforts being essential for developing most widely used FST methodologies.^30^ Thus, the suitability of applying metagenomic approaches to FST is difficult to ascertain.^31^ Moreover, results from metagenomic sequencing and bioinformatic analysis can be difficult to confidently interpret in an FST context, having unclear or untested relationships with microbial, and thus fecal, loads.^31–36^ In total, these limitations fail to provide users with certainty regarding when it is appropriate to employ metagenomic FST, whether it offers any benefits compared to established methods, and how the choice of parameters or upstream bioinformatic processing impacts performance for their system.

In response to these limitations, we theorized and tested a new framework for metagenomic FST based on read mapping of metagenomic data to source-associated genome databases and normalization by meaningful abundance units i.e., genome equivalents,^32^ to approximate cell fractions. In contrast to the methodology of existing approaches, we opted to leverage curated genome databases so that analysis could be performed without one needing to sequence potential sources. We investigated the strengths and limitations of our framework via experiments utilizing shotgun sequencing of fecal spike-in freshwater mesocosms of known composition. Lastly, we integrated this framework within a new tool called SourceApp to streamline source database curation and metagenomic FST analysis, enabling researchers to apply our approach to fecal sources relevant across diverse monitoring sites and study designs.

## 2 Materials and Methods

We performed a study benchmarking the performance of metagenomic FST with lake water mesocosms containing fecal or synthetic whole-cell microbiome spike-ins. The prokaryotic cell densities of lake water and fecal samples were approximated by microscopy to calculate cellular ratios after spiking in. The mesocosms were created using lake water and fecal contamination sources relevant to the study (*n* = 32 mesocosms and 8 source categories) as well as a commercially available synthetic microbial community (ZymoBIOMICS microbial community standard D6300) (*n* = 3). All mesocosm samples were shotgun sequenced using Illumina NovaSeq and fecal contamination sources were sequenced using both Illumina NovaSeq and Oxford Nanopore Flongles. The mesocosm short read sequence data was used to perform common FST exercises: source attribution and apportionment. Synthetic community standards were used to assess the accuracy of our methods and assist with calibrating total cellular load estimates.

### 2.1 Spike-In Preparation

For our mesocosm experiments, we collected approximately 20 L of lake water from Lake Lanier, Georgia, (34°15′20″ N −83°56′40″ W) in sterilized, acid-washed plastic carboys in May 2022. We used a Colilert (IDEXX, Norcross, GA) assay to quantify the concentration of E. coli in the lake water samples to confirm FIB levels did not exceed Recreational Water Quality Criteria before continuing. Lake water (100 mL) was assayed using this method within 24 h of collection.

Sources of fecal contamination used in this study included: sewage, septage, cow, pig, dog, cat, chicken, and goat feces. Fecal samples from these sources were collected as previously described.^37^ In brief, we collected 1 L of 24 h composited primary influent samples from three water reclamation facilities (WRF) in Georgia, as well as 500 mL of composite septage samples, in sterilized, acid-washed plastic Nalgene bottles. For our animal fecal inputs, we collected at least 10 individual feces samples from each animal fecal source studied (cow, pig, dog, cat, chicken, goat). All samples were stored on ice in the dark during transport to the lab and were processed within 24 h of collection.

In the lab, the 10 individual fecal samples (1 g per individual) for each animal type were combined to make a fecal slurry for each. Equal masses of fecal samples were composited in a sterile 50 mL conical tube and filled to a volume of 50 mL using sterile filtered 1X PBS. Slurries were kept at 4 °C in the dark until cell counting, which took place within 48 h of sampling. Leftover fecal slurries were diluted 1:3 in Zymo DNA/RNA Shield (Irvine, CA, cat. no. R1100-50) and stored at −80 °C until extraction.

### 2.2 Fluorescence Microscopy

Microscopy-based cell density measurements for lake water, sewage, septage, and animal fecal slurries were completed using 4′,6-diamidino-2-phenylindole (DAPI) cell staining and imaged on a ZEISS Axio Observer D1 (Oberkochen, Germany). Additional information for the microscopy methods used can be found in the Supporting Information. Cell density estimates were used to ensure appropriate volumes of fecal slurry could be spiked in to achieve desired cellular fractions within the experimental mesocosms.

### 2.3 Mesocosm Construction

Within 48 h of sample collection, mesocosms were mixed in sterilized, acid-washed glass beakers at a volume of approximately 500 mL (lake water) per mesocosm. Volumes of each fecal slurry to add to each mesocosm were calculated by the following equations

1 $$total\;cells=\sum_{i=1}^{n}\left(\rho_{i}V_{i}\right)+\rho_{lake}V_{lake}$$

2 $$fecal\;{source}_{i}\;cells=\rho_{i}V_{i}$$

3 $$fecal\;{source}_{i}\;cell\;fraction=\frac{fecal\;{source}_{i}\;cells}{total\;cells}$$

where ρ represents cell density for the background matrix, a fecal slurry, or sewage sample (cells/mL). The maximum and minimum number of fecal sources (*i*) mixed in a single mesocosm was six (*n* = 6) and one, respectively, excluding the negative control which was constructed with only lake water.

For mesocosm sample processing, beakers were mixed with a pipet tip to stir and were filtered within 30 min of mixing. Sterile 0.22 μm pore size, 47 mm diameter membrane filters (Sartorius, Gottingen, Germany, cat. no. #11407-50-ACN) were placed on the base of a sterilized, acid-washed magnetic filter funnel using flame sterilized forceps for each mesocosm type. A 200 mL aliquot of each mesocosm was filtered onto a membrane, in duplicate, using vacuum filtration and sterile filtered 1X PBS was used to rinse the sides of the filter funnel. Filter membranes were folded and placed into sterile 2 mL screwcap tubes and researchers were blinded from the mesocosm types using a random three-digit code. Tubes were frozen at −80 °C until nucleic acids were extracted, approximately three months later. A concentration step blank was created by passing filter sterilized 1X PBS through a membrane in the same manner as the other mesocosms.

Aliquots of each fecal slurry or source were archived after all mesocosm work was completed. Fecal slurries were diluted 1:3 in DNA/RNA shield (Zymo, Irvine, CA) and 8 mL was stored in screwcap tubes at −80 °C for six months before extraction. For sewage samples, the sewage was membrane filtered and stored at −80 °C for six months before extraction.

### 2.4 DNA Extraction and Sequencing

Filter membranes with mesocosm biomass were extracted using the Qiagen DNeasy PowerSoil Pro kit (Hilden, Germany, cat. no. 47014) following the manufacturer’s instructions on a Qiacube Connect instrument. The fecal slurries used to inoculate the mesocosms were extracted using Zymo’s Quick-DNA HMW Magbead kit (cat. no. D6060) following the manufacturer’s instructions. Purified DNA extracts from the mesocosms were shotgun sequenced on the Illumina NovaSeq 6000 instrument for 2 × 150-bp reads. Illumina sequencing of fecal slurries was performed similarly, as described previously.^37^ The high molecular weight DNA extracts from the mesocosms were sequenced on an Oxford Nanopore minION using Flongle flow cells (Oxford, UK, cat. no. R10.4.1). Additional information on DNA extractions, library preparation, and sequencing can be found in the supporting documentation.

### 2.5 Assembly and Binning of Fecal Spike-In Material

Short and long reads were used to recover MAGs from fecal slurries, wastewater, and septage samples. Two approaches were taken to recover high quality genomes from the fecal spike-in sequence data produced: First, a short read only approach. Second, a hybrid approach combining short and long reads.

For the short-read approach, the sequences were quality checked using fastp^38^ to remove leftover adapter sequences and low-quality reads. Trimmed reads were assembled using metaSPAdes.^39^ In parallel, trimmed reads were normalized with BBtools’ bbnorm (“target = 30”, “min = 5”) and assembled using metaSPAdes but without census correction via BayesHammer (e.g., --only assembler).^40^ The resulting assemblies, from both trimmed and normalized reads, were binned with both MaxBin and MetaBAT to produce MAGs.^41,42^ MAG quality was assessed with CheckM2 ^43^ and genomes with aggregate quality scores below 50% as calculated by quality = completeness −5 × redundancy were discarded.

For the hybrid approach, the base called long read sequences were quality checked with NanoPlot (for visualizations only) and trimmed with filtlong (--min_length 1000 --min_window_q 0.8 --window_size 250). The trimmed long reads and trimmed short reads were then assembled with metaSPAdes (using --nanopore).^39^ Hybrid assemblies were binned, and the resulting MAGs were checked for quality as with the short-read approach above.

All medium and high-quality MAGs were dereplicated (at 95% ANI) within their source category to select a single best representative for a species-cluster using dRep (using default settings except --S_algorithm fastANI -comp 50).^44,45^ MAG taxonomic classification was determined against GTDB (release 220) using the classification workflow of GTDBtk (v2.4.0) via default parameters.^46,47^

### 2.6 FST Exercises: Attribution, Apportionment, and Cell Fraction Estimations

To facilitate source attribution and apportionment exercises, we constructed databases of prokaryotic genomes with known source associations. An environmental category was also created by including genomes from large sequencing studies of the freshwater environment.^17,48^ For fecal sources, we used genomes from publicly available data sets previously collected and curated into a database by our team.^37^ Additionally, we augmented this database with the MAGs recovered from assembly and binning of the short and long read fecal slurry metagenomes, similarly dereplicated within their source categories as described above. Genome pairs in separate source categories were flagged as cross-reactive when ANI values greater than or equal to 95% were observed (Table S2).

Mapping of short reads from the mesocosm metagenomes to the source databases described above was the core component of the source attribution, apportionment, and cell fraction estimation exercises. Read mapping was accomplished with the bwa (mem) algorithm although other read mapping algorithms were also tested.^49^ Sequence depth and number of reads mapped to genomes in each source category were determined via CoverM (v0.7.0; https://github.com/wwood/CoverM). Various parameters were explored for read mapping and sequence depth estimation and are detailed in the next section as part of parameter tuning efforts.

Cell fractions were estimated by summing the sequence depth of all genomes in a source category and then normalizing to genome equivalents as estimated by MicrobeCensus.^50^ This estimates relative abundance in terms of genome copies to total genome copies (i.e., genome equivalents or GEQ). Similarly, the number of reads mapped to each genome in a source category was summed and normalized to the total reads in a metagenome to calculate read-based relative abundance. Source apportionment was accomplished by further normalizing the signal from each source category by the total detected fecal cell fraction (i.e., the sum of the relative abundances of signal from each source in the database).

Reporting of source attribution and apportionment in these experiments utilized the following logic: for attribution, to consider a source “present” at least two conditions must be met. First, at least one source-specific genome must be detected in the metagenome. Second, the estimated cell fraction for a source must exceed the parameter set for limit of detection (0.01% by default). For apportionment, to estimate the portion of fecal signal belonging to a source it must first be considered “present” according to the attribution conditions described above. If so, then that source’s portions were estimated as the sum of both source-specific and cross-reactive cell fractions divided by the sum of all “present” sources.

In summary, for a given source *j*, the following relationships demonstrate how source portions and cell fractions were estimated

4 $$cell\;{fraction}_{j}=\sum_{i=1}^{n}\left(\frac{X_{i}}{GEQ}\right)$$

5 $$source\;{portion}_{j}=\frac{cell\;{fraction}_{j}}{\sum_{i=1}^{k}\left(cell\;{fraction}_{i}\right)}$$

where *X* is a genome’s sequencing depth, *n* is the number of genomes belonging to a source category (both specific and cross-reactive), and *k* is the number of source categories present in the database.

Ground truth values were established during the mesocosm construction as described above and used in scoring attribution and apportionment. These values are recorded in Supporting Information (Table S1).

### 2.7 Evaluation, Parameter Tuning, and Scoring

Source attribution and cell fractioning exercises were iterated across 640 combinations of bioinformatic parameters. Greater details on the chosen parameters, their ranges, functions used in scoring, and parameter selection can be found in Supporting Information.

Following parameter optimization, we evaluated the performance of SourceApp against alternative tools for the same purposes: metaSourceTracker,^24^ FEAST,^23^ and decOM.^25^ Taxonomic profiles were generated using Kraken2 ^51^ with default settings for both mesocosm and fecal slurry metagenomes. For decOM, *k*-mer count tables were generated with kmtricks^52^ as instructed in the decOM documentation. Further information on tool-to-tool comparisons can be found in the Supporting Information.

## 3 Results and Discussion

### 3.1 SourceApp Development and Database Construction

We developed and tuned the parameters of a bioinformatic pipeline we call “SourceApp” to fully automate the methods used in this work. SourceApp is easy to use, has transparent parametrization, and allows practitioners to focus on sources relevant to their systems both by supporting custom database creation and facilitating end-to-end processing of metagenomic data from raw reads to results (i.e., source attribution, apportionment). Additional information on the development of SourceApp, including the composition of its genome databases, can be found in the Supporting Information (SI Methods, Table S2) and online user documentation (https://github.com/bglindner/SourceApp).

For the results discussed in subsequent sections, all signals from human-specific genomes have been aggregated within the wastewater category but flagged as cross-reactive in SourceApp’s source attribution logic. Lastly, when genomes representing the environmental matrix were included as a “screen”, the signal mapping to these genomes was excluded from source apportionment as the goal was to produce source portions that correspond to the portion of fecal contamination belonging to each source. These operations, among several others, are adjustable by the user when running SourceApp and fully detailed in both the user documentation and Supporting Information.

### 3.2 Source Attribution and Apportionment

Data from all mesocosms containing fecal spike-ins (*n* = 31) and the negative control mesocosm containing only lake water (*n* = 1) were processed with SourceApp and reports of source attribution were collected. Following parameter tuning, optimal source attribution results were achieved with the following parameters: 93% minimum read-to-genome alignment identity, 70% minimum alignment-to-read overlap, 0% target genome masking, and a limit of detection (LOD) of 0.01% relative abundance, which yielded 0.898 specificity and 0.902 sensitivity (Figure S1; Supporting Information). Other parameter sets scored similarly as performance saturation was observed with multiple sets of similar parameters (Table S3). For example, minimum alignment-to-read overlap was the least impactful parameter on source attribution performance with top-scoring sets containing all tested values for this parameter (30–90%). In contrast, all top-scoring sets contained 93%–95% as minimum alignment identities consistent with the importance of this parameter for properly identifying reads originating from organisms of the same species as the reference genome.^53^

Since source apportionment relies on accurate attribution, we performed apportionment using the best scoring attribution parameters as identified above and visualized in Figure 1. Across the 32 mesocosms, most contained some amount of wastewater input with specific information on the spike-in amounts noted in between the upper and lower panels of Figure 1. Most sources were correctly attributed across all mesocosms including true negative calls for all categories in the negative control sample. Source categories for which SourceApp’s predictions were most accurate included wastewater, septage, cat, and dog. Sources for which poor performance was observed were limited to specific sources, namely cow, pig, chicken, and ruminant. For example, all instances of fecal signal being falsely attributed were to the pig (*n* = 19) or chicken (*n* = 1) source categories. Yet, for the mesocosms containing only wastewater (e.g., those labeled WRFA, B, or C and which belonged to different WRF), we observed pig false positives only when the cellular concentration of the wastewater spike-in was quite high (10% c/c). This observation was consistent across the raw influent collected from three different WRFs (i.e., A, B, and C). Possible explanations for this observation include pig feces input into these sewersheds or, more likely, the presence of species associated with both pig and wastewater, but which have currently only been captured in the pig database. We discuss the latter hypothesis in greater detail below.

Despite efforts to increase the number of source-associated genomes in the cow database, all mesocosms containing cow spike-in signal were reported as false negatives. Previous analysis of the fecal slurry prepared for the cow spike-in revealed it was substantially more diverse than even its nearest neighbor (goat/ruminant) in terms of expected sequence diversity, suggesting a substantial number of reference genomes would be needed to capture cow signal.^37^ For example, the goat database contained 759 species compared to the 43 species currently in the cow database (Table S2).

**Figure 1:**
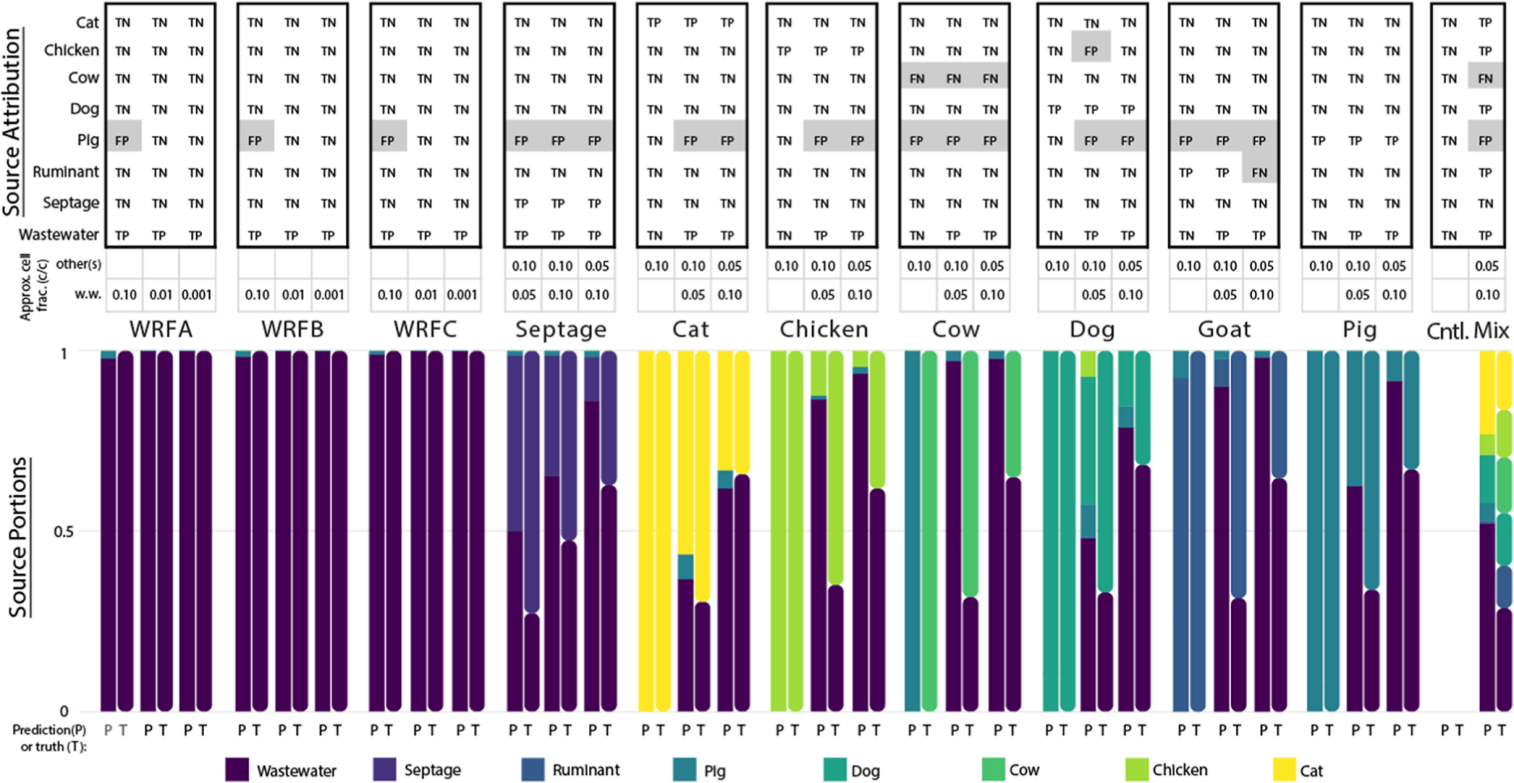
Results of source attribution and apportionment of fecal spike-in metagenomes and negative control with SourceApp. The results shown are sorted column-wise and results for mesocosms are grouped together by the type(s) of spike-in they received (see color key at the bottom). False attributions are shaded gray for convenience (top). Each pair of bars depicts the predicted (“P”, left square bars) and true (“T”, right rounded bars) source portions for a sample according to cell density estimates collected during mesocosm construction, where true “source portions” were calculated as described above in eq 5. All mesocosms receiving any amount of wastewater spike-in received wastewater collected at WRFA unless labeled explicitly as WRFB (*n* = 3) or WRFC (*n* = 3). Note the approximate cell fraction (c/c) which describes the final cellular concentration of wastewater and/or other sources (if present). Cellular fractions shown are based on the cell density estimates for the environmental matrix and fecal slurries used when constructing each mesocosm as described in the methods and are not volumetric fractions (Table S1). The “Mix” mesocosm was constructed to achieve final cellular concentrations of 10% wastewater associated-cells and 5% each of cat, goat, chicken, cow, and dog feces associated-cells. TP = true positive, FP = false positive, FN = false negative and TN = true negative, WRF = water reclamation facility.

Additional details on positive reporting by SourceApp are summarized in Figure 2. Overall, false positive calls made by SourceApp were based on lower signal than true positive calls. For example, most false positives were based on detection of fewer genomes (mean = 5.4) than true positive calls (mean = 36.1) and represented much smaller portions in the subsequent apportionments (Figure 2). As suggested above, false positive calls from pigs primarily resulted from repeated detection of reads mapped to only a few species of the pig database (Figure S2 and Table S4). The negative control (“Cntl.”) contained no false positives from any source category as shown above in Figure 1. Wastewater, the most ubiquitous and often highest concentration spike-in source category, was never falsely identified among the source attribution results. False negative attributions by SourceApp belonged to cow (*n* = 4) and ruminant (*n* = 1) source categories.

**Figure 2:**
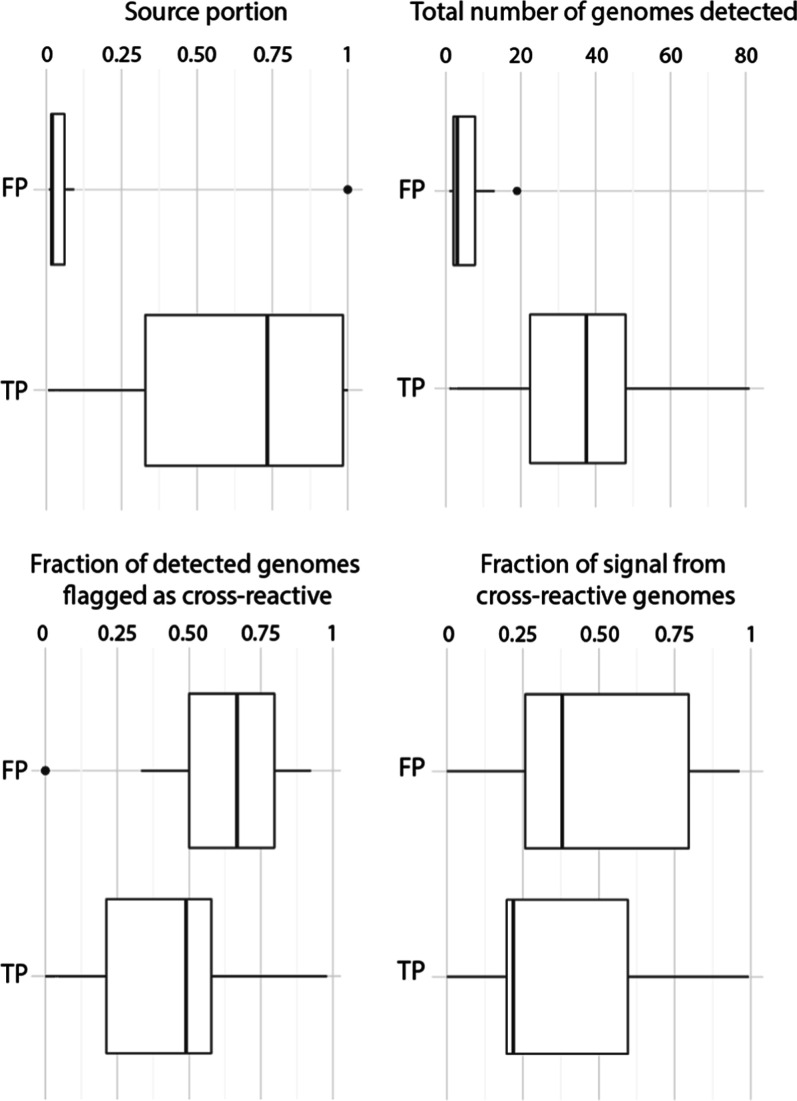
Characteristics of true and false positive predictions by SourceApp in terms of number of database genomes detected. Most false positive predictions by SourceApp constituted very small portions in the subsequent source apportionment. False positive calls were usually based on the detection of substantially fewer genomes (mean = 5.4) than true positive calls (mean = 36.1). Genomes flagged as cross reactive tended to make up a larger percentage of the total detected genomes for false positive calls (mean = 57%) compared to true positives (mean = 46%). The fraction of signal (i.e., relative abundance) contributed by cross reactive genomes varied substantially for both true and false positive calls.

Using the ideal parameters reported by parameter tuning efforts above, we also performed the source attribution exercise against the primary database constructed without an environmental genome set (see Table S2). Performance was worse across all metrics when using databases without environmental genomes, particularly in the case of wastewater attribution and apportionment, illustrating the importance of supplementing genome collections used for database construction with site specific genomes and the need for inclusion of genomes associated with the environment to filter cross-reactive signal from autochthonous microbes. Specifically, when comparing these results to those shown in Figure 2, the inclusion of environmental genomes reduced the occurrence of false positives in source attribution by about a quarter (from 27 to 20; Figure S3).

To better contextualize SourceApp’s performance, we used FEAST, metaSourceTracker, and decOM to analyze metagenomes from both the mesocosms and sources (Tables S5 and S6). Generally, we found that each tool implicated the presence of all possible contaminating sources regardless of whether a tool was based on taxonomic profiling via Kraken2 (e.g., FEAST and metaSourceTracker) or *k*-mer profiling (decOM). In terms of source attribution, this resulted in correct identification of all true positive cases of contamination (i.e., sensitivities of 1) but poor specificity due to a preponderance of false positives—with specificity values of 0.30 for FEAST and 0 for both decOM and metaSourcetracker. High false positive rates remained despite our efforts to implement a minimum reported portion to call sources present (i.e., an LOD), and suggests the need for end users of these tools to develop their own heuristics when interpreting each tools’ results (Table S7). In contrast, SourceApp has slightly lower sensitivity but far improved specificity suggesting its ability to improve the interpretation of metagenomic data in an FST context. This is further supported by SourceApp’s source apportionment performance; correlation coefficients between each tool’s results and the ground truth were 0.86, 0.79, 0.74, and 0.60 for SourceApp, FEAST, decOM, and metaSourceTracker, respectively (Table S8). These benchmarking efforts resolve the efficacy of SourceApp and confirm reports by others of better FST performance with FEAST than SourceTracker^54^ and the difficulty of resolving mixed input fecal contamination—particularly when biogeography influences site specific fecal community composition – by tools developed heretofore.^28,29^

Metagenomes from the synthetic microbiome spike-in mesocosms were analyzed and portions estimated for each species before comparison to the ground truth reported by the manufacturer. Observed error between species-level estimated and expected portions varied with taxonomy and rank abundance. For example, disparities between predicted and expected species portions tended to be greater among lower ranking species across all spike-in cell fractions (Figure S4).

### 3.3 Estimating Fecal Associated Cell Fractions

Estimations of total fecal load are crucial to FST methodologies for distinguishing between magnitudes of fecal pollution. In the context of this work, we used estimates of cell fraction to infer the intensity of fecal pollution in each mesocosm. The results from each iteration of cell fractioning parameter tuning are recorded in Supporting Information (Table S9). Cell fractions estimated by SourceApp using the best performing parameter set ranged from 0 to 7% (c/c) for each source and are reported in Figure 3.

**Figure 3:**
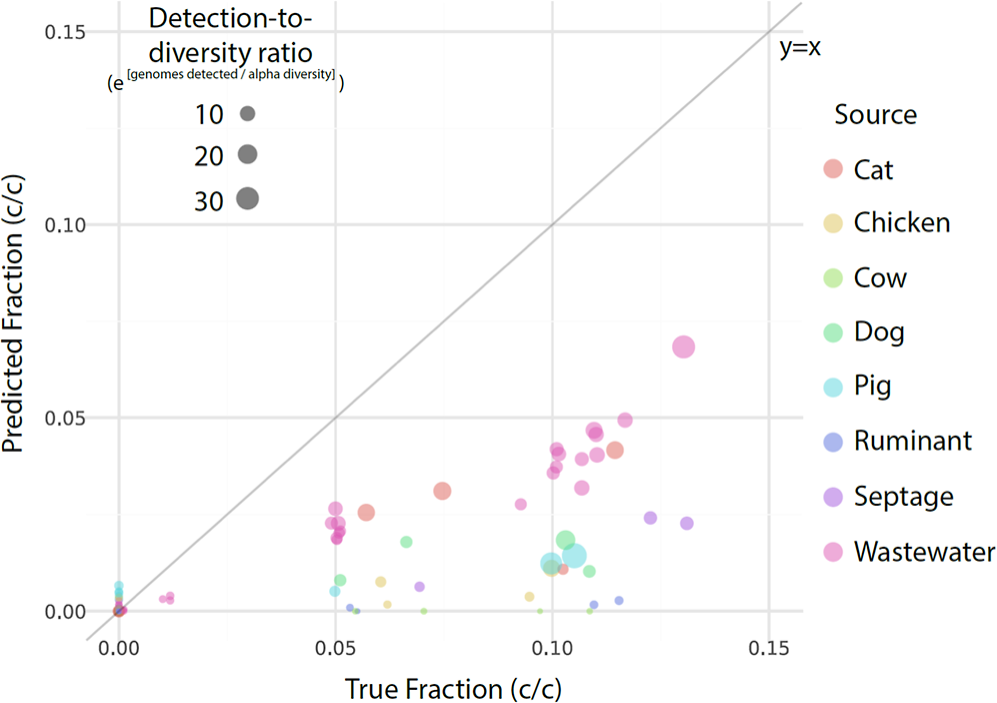
Cell fractions predicted by SourceApp for fecal spike-in mesocosms compared to ground truth cell fractions calculated by microscopy at mesocosm construction. All sources have 32 points representing the values predicted for each source’s cell fraction within all mesocosms (*n* = 32). False positives occur along *y* = 0, false negatives along *x* = 0, and true negatives at the origin. The size of each point is scaled to the exponential of the ratio formed by the number of genomes detected to the estimated alpha diversity of the fecal spike-in metagenome; thus, larger points indicate more genomes were detected relative to the fecal material’s alpha diversity. Note the consistent trend of increasing point diameter up and to the right, indicating that as coverage of a source’s diversity increases within SourceApp’s database, cell fraction estimates increase in accuracy. The scale of true and false negative points is not always 1 (i.e., e^0^) since cross-reactive genomes can be detected but disregarded by SourceApp’s logic if at least one source specific genome was not also detected.

SourceApp consistently underestimated cell fractions by a factor of 2 to 3. Accordingly, the slope reported by the linear regression prepared in the parameter tuning exercise was 3.4, although substantial residuals remained after linear correction even with the best performing parameters (*r* ^2^ = 0.64; Figure S5). This finding was consistent with the synthetic spike-in microbiomes; SourceApp’s methodology apportioned the 10 populations into accurate portions in the synthetic microbiome spike-ins across all dilutions, but the associated estimation for total cell fraction belonging to the synthetic microbiome members were similarly underestimated even when controlling for environmental screening in the genome database. In fecal spike-in mesocosms, cell fraction estimation varied across sources, with wastewater, septage, and cat signals appearing most consistent while other sources with databases that failed to effectively capture fecal signal appearing worse (Figures 3 and S6). For example, across mesocosms containing approximately 5% (c/c) spike-in cellular concentrations, SourceApp predicted cell fractions for wastewater (which has the most comprehensive genome database) higher than predicted cell fractions for cow, pig, ruminant, etc.

The concordance between synthetic and fecal spike-in mesocosm cell fraction predictions and the disparity between wastewater and poorly covered source categories suggest two associated conclusions. First, that some amount of systematic error in the experiments is driving cell fraction underestimations across all mesocosms and sources but with essentially no impact to source proportions. It is worth noting that a similar slope (∼3) was found after linear correction when examining the cell fraction estimates returned by SourceApp from among all synthetic microbiome spike-in samples, suggesting that the source of error may be underestimation of the background cell concentration (i.e., lake water) by the microscopy methodology since this matrix was common across all samples (Figure S7). The second conclusion, in conjunction with the first, is that as genome databases for source categories grow and read capture rates improve, the ability to calibrate metagenomic results for accurate cell fraction predictions should improve while also concomitantly preserving the integrity of source apportionment results.

### 3.4 Environmental Relevance

One of the primary goals of FST is to assist with managing waterbodies that are out of regulatory compliance due to fecal pollution. Inferring the source(s) of fecal contamination in impaired waterbodies—especially relative contributions from among multiple offending sources—is a challenging task. Selection of the best tool for this task from within the ever-growing toolbox of FST techniques can be daunting when the end goal is actionable guidance for remedial efforts or public health decisions.^16,55^ To our knowledge, no work exists which has benchmarked metagenomic FST methodologies against gold standard data sets such as the spike-in mesocosms constructed herein. Thus, the present work represents the first effort to explore how well shotgun metagenomic data can elucidate instances of mixed fecal contamination, including crucial FST goals like source attribution and apportionment. Our initial findings suggest that for sources with sufficiently well-curated source-associated prokaryotic genome databases, it is possible to accomplish source apportionment with shotgun metagenomes. Further, we found that the genome databases used may not necessarily need to be quite large (in terms of the number of genomes accessioned) to effectively capture signals depending on the underlying microbial diversity of a fecal source (Figure 3). For example, all mesocosms containing mixtures of wastewater and septage were apportioned reasonably well despite the septage source-associated genome database containing only 39 MAGs produced herein (Figure 1 and Table S2).

Of the sources examined, the majority could be correctly attributed across a range of cellular spike-in densities and even in mesocosms of mixed inputs. Our mesocosm experiments, some of which were spiked with a synthetic microbiome instead of fecal material, confirmed that SourceApp accurately apportioned sources across a broad range of input concentrations even when cell fractions were underestimated; improving source attribution led to source apportionment predictions that matched the ground truth, independent of cell fraction estimates. False positive attribution, particularly for the pig source category, occurred in several samples but the signal associated with these calls suggested the root cause was cross-reactivity with only one or two species shared between sources (Figures 1 and S2). This highlights the need for growing genome databases—a major requirement for SourceApp’s efficacy. In a similar vein, false negatives for cows across all samples point to a poor understanding of the prokaryotic content of cow feces at the genomic level, contrasting with cow rumen communities that have been well sampled but are largely different from the hindgut fecal communities.^56^ In these circumstances, should those interested in using metagenomic sequencing for FST be interested in tracking cow fecal contamination, plans to sequence samples of the source feces itself to recover MAGs as supplements for the existing database will be necessary.

As part of this work, we have presented SourceApp and updated our recently described FST whole genome database^37^ to establish a framework for comparing metagenomic FST efforts across different research teams and scenarios—particularly for those interested in source apportionment aims. We found that including genomes representing local or site-specific prokaryotic populations within the database used by SourceApp improves performance when compared to using genomes derived from the literature alone by both improving sensitivity to fecal sources and controlling for cross-reactivity with environmental populations. Thus, users striving for optimal SourceApp performance should update the reference genome databases we have initialized, when possible, with additional genomes from potential offending sources (either by isolate sequencing or binning shotgun metagenomic data). Toward this end, the databases produced for use with SourceApp have been made publicly available for download and are hosted on the MiGA Web server^57^ where users can upload genomes from their own work to explore matches within and across source categories. Lastly, SourceApp’s ability to autonomously create new databases (including new source categories within them) when provided genomes by the user can allow investigators working across diverse geographies and watershed characteristics to utilize the metagenomic FST methods developed herein for use with their own data sets derived from local sites. Overall, these efforts inform FST practitioners about the effectiveness of metagenomic FST methods across a range of sources or cell fractions and can assist with processing and interpreting metagenomic FST via the new SourceApp tool.

### 3.5 Limitations and Future Perspectives

This work explored two components of metagenomic analysis which could impact the efficacy of metagenomic FST. These analytical components included genome databases of differing composition (Table S2) and sets of various parameters commonly used to interpret read mapping results (Table S3). Based on the results of iterating hundreds of SourceApp runs across these different genome databases and read mapping parameters, we discerned effects on performance related to both. To ameliorate these effects, the objective function used to tune SourceApp’s attribution capabilities and recommend default parameter choices was based on maximizing the negative predictive value for results across all fecal spike-in mesocosm experiments. Thus, the results for attribution (Figure 1) reported here achieve the maximum observed value for sensitivity—yet not specificity—across the entire parameter tuning exercise (Figure S1). Our work suggests that achieving better performance from this type of metagenomic FST not only relies on good parameter selection but on constructing more robust genome databases—especially for sources with poor attribution results (e.g., cow, ruminant, pig)—in the future.

Additionally, despite implementations of a minimum cell fraction threshold for source apportionment in the parameter tuning exercise, the distribution of estimated cell fractions for both false positives and true positives overlapped sufficiently that no single threshold value could effectively eliminate false positives without also discarding many true positives. This was due in part to low dosages for some of the spike-in material in certain mesocosms (e.g., 0.1% c/c; Table S1) but largely resulted from uncontrolled cross-reactivity between the same species found in differing sources being detected across multiple mesocosms (e.g., the pig source category; Figure 2). Though, many of these false calls were linked to specific populations which suggests avenues for future work to address this challenge (Figure S2). When examining the proportions of each source in the apportionment step, false positives were usually at or below portions of 5–10%. This highlights the capacity of this tool and its multiple outputs to inform the end user in a way that aids the interpretation of results: sources reported at very low proportions could be flagged or considered spurious (i.e., representing false positives). Yet, these results were not without error which was substantial in certain cases (Figure 1).

The species-level representative genomes used as markers by SourceApp represent population with largely unknown persistence in the environment. This means that at this time, SourceApp cannot infer the age of contamination or elucidate which signal is from nonconservative populations (i.e., capable of growth in the environment). Future experiments benchmarking metagenomic FST methods should incorporate investigations of aging both to assess aged fecal signal’s impact on the results of tools like SourceApp and to determine appropriate methodologies to overcome it.

Lastly, the portions reported by SourceApp do not represent the results of a mass balance but rather efforts to estimate the proportions of cells belonging to source categories based on genome equivalents. That is, the estimations for cell fractions made by SourceApp may not reproduce the relative inputs from each fecal source in terms of mass inputs (should this information be known to the user). Therefore, depending on the cellular density of each fecal source, the portions represented here will vary from what one would expect based on a mass balance analysis.^3^ Though SourceApp’s results could be used to perform source apportionment to reproduce mass inputs if a reliable estimate for the average cell density of suspected fecal inputs is known. Despite these caveats between cellular fractions and mass inputs, units with a genomic basis are important for metagenomic FST because they enable tools like SourceApp to reproduce portions and cell fractions with relative accuracy as we have shown. Indeed, this study represents an important evaluation of the use of genome equivalents as the basis for reproducing ratios between biological units in metagenomic data sets—something that has been proposed as a “universal unit” for use across the field by others but rarely benchmarked as done here.^32^

## Supplementary Material





## Data Availability

All code developed for this work, including the SourceApp tool, and code used for parameter tuning, is freely available online with supporting documentation at https://github.com/bglindner/SourceApp. All sequence data from the fecal slurries and mesocosms has been uploaded to NCBI under BioProjects PRJNA1092107 (spike-in slurries) and PRJNA1161616 (mesocosms). The primary genome database (with environmental screen) constructed for use with SourceApp has been indexed for querying user genomes and is fully browsable online at the Microbial Genome Atlas (MiGA) Web server (https://uibk.microbial-genomes.org/projects/SourceApp).^57^ The genome database has also been packaged for download on Zenodo (10.5281/zenodo.12795076) where it can be obtained for immediate use with SourceApp.

## Notes

Additional methodological details and graphics summarizing SourceApp development, parameter tuning, and benchmarking (Figures S1–S8) (PDF)
Supporting Information describing experimental design, database curation, SourceApp performance, and tool-to-tool comparisons (Tables S1–S9) (XLSX)
