# Supplementary material for: SourceApp: A Novel Metagenomic Source Tracking Tool that can Distinguish between Fecal Microbiomes Using Genome-To-Source Associations Benchmarked Against Mixed Input Spike-In Mesocosms
Source: Environ Sci Technol. 2025 May 6;59(19):9507–16. doi: 10.1021/acs.est.5c03603 (PMC12101495; doi:10.1021/acs.est.5c03603)
Supplement: Supplementary file 1 [file es5c03603_si_001.pdf]

SUPPORTING INFORMATION FOR

SourceApp: A novel metagenomic source tracking tool that can distinguish between fecal microbiomes using genome-to-source associations benchmarked against mixed input spike-in mesocosms

Blake G. Lindner[1], Katherine E. Graham[1], Jacob R. Phaneuf[1], Janet K. Hatt[1], Konstantinos T. Konstantinidis[1,2,*]

[1] - School of Civil and Environmental Engineering, Georgia Institute of Technology, Atlanta, GA 30332, USA.

[2] - School of Biological Sciences, Georgia Institute of Technology, Atlanta, GA 30332, USA.

[*]Corresponding author: Konstantinos T. Konstantinidis (kostas@ce.gatech.edu)

Number of SI Pages: 15
Supporting Figures: 8
Supporting Text Sections: 5
Supporting Tables: 9

TABLE OF CONTENTS

**Entry**	Page Number
**SI Figures**	
Figure S1. Parameter tuning of SourceApp for source attribution	S3
Figure S2. Summary of taxonomic classification of all detected pig-associated genomes across samples with false positives	S4
Figure S3. Confusion matrices illustrating SourceApp performance with and without environmental genomes from aquatic prokaryotes	S5
Figure S4. Results from analyzing metagenomes obtained from the three synthetic community (Zymo) spike-in mesocosms with SourceApp against the databases built without (top) and with (bottom) an environmental genome set (for genome database information see Table S2)	S6
Figure S5. Parameter tuning results for use of SourceApp for cell fractionation	S7
Figure S6. Fecal cell fractions across all sources and all mesocosms after correction with the slope (m) of the linear model producing least residuals found via parameter tuning	S8
Figure S7. Total cell fraction predicted end expected values for the three synthetic spike-in mesocosms	S8
Figure S8. Workflows associated with SourceApp's two main subroutines	S14
**SI Text**	
Fluorescence Microscopy	S9
DNA extraction and sequencing	S10-S11
Parameter tuning and scoring	S11-S12
Tool-to-tool Comparisons	S12-S13
SourceApp Development and Database Construction	S13-S15
SI Citations	S15
**SI Tables**	
Tables S1-S9 – see spreadsheet	.xlsx

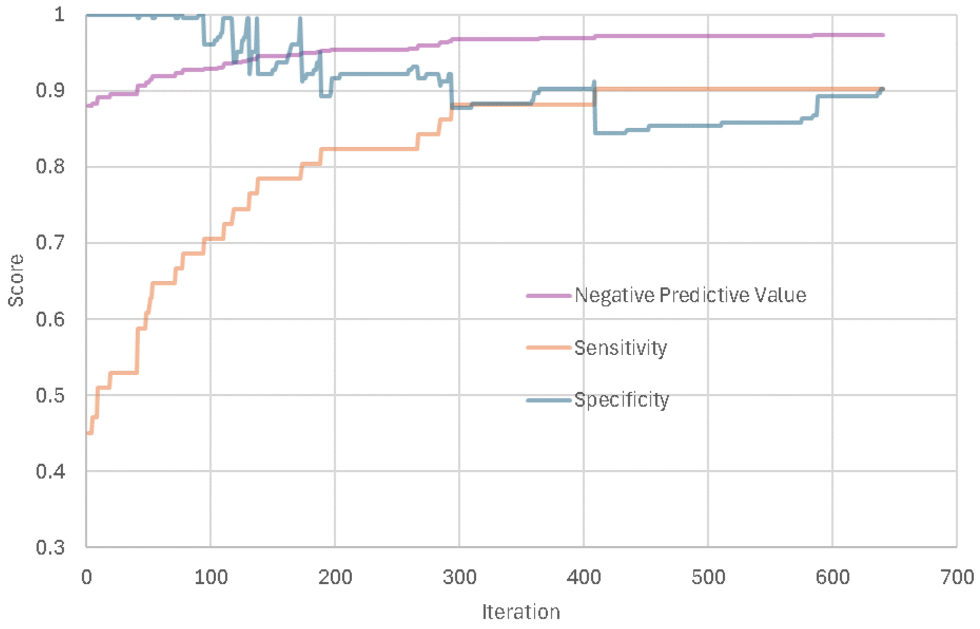

Figure S1. Parameter tuning of SourceApp for source attribution. Over 600 different parameter combinations were attempted and efforts to tune the software were driven by maximizing the negative predictive value. See also Table S2 and supporting information for further information. Sensitivity represents the true positive rate, or the probability that SourceApp reports a true positive correctly. Specificity represents the true negative rate, or the probability that SourceApp reports a true negative correctly. The negative predictive value is the proportion of negative calls reported which are true (>90%).

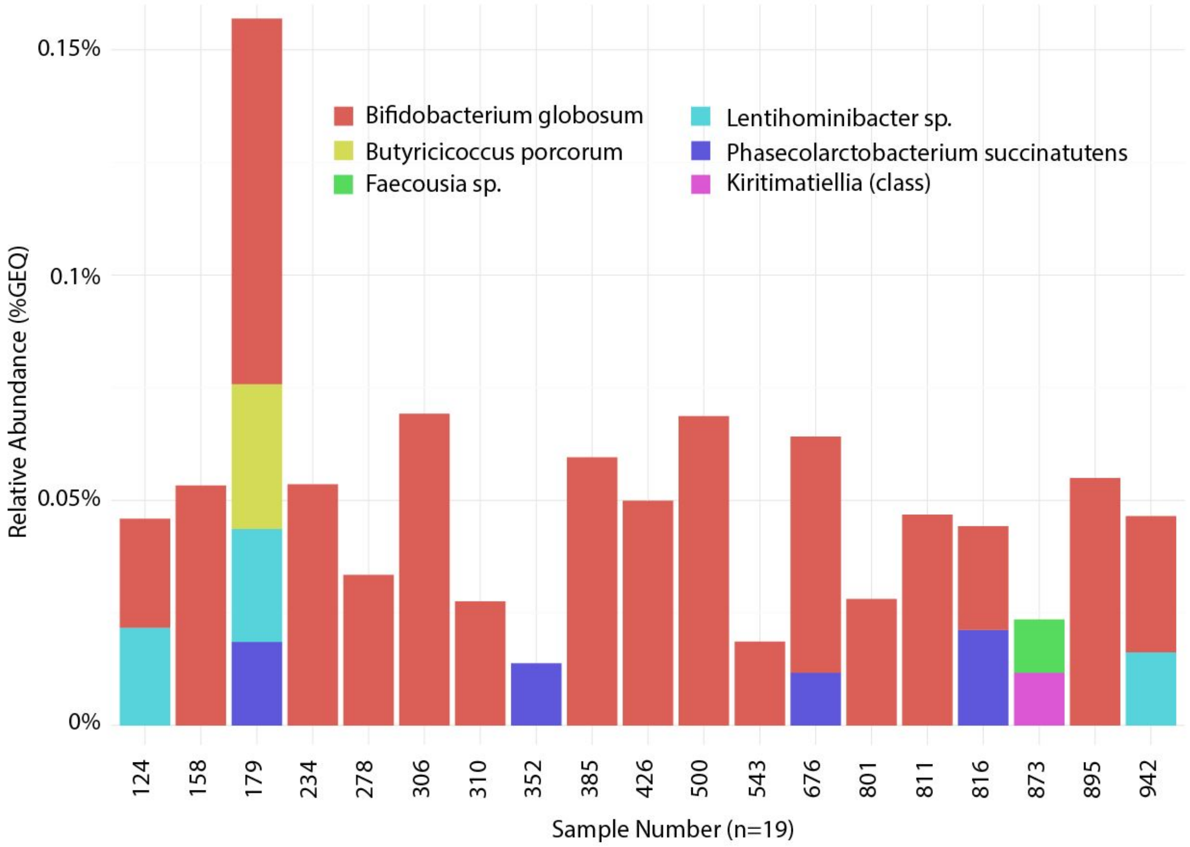

Figure S2. Summary of the taxonomic classification of all detected pig-associated genomes across samples with false positives. Each x-axis index (e.g., 124, 158, etc.) represents a different mesocosm. The taxonomic composition of these false positives was restricted to only a few species, primarily *B. globosum*. See also Table S3 for further details and Table S1 for spike-in composition within each mesocosm.

		Contains environmental genomes		Missing environmental genomes	
MAGs + literature review		Predicted		Predicted	
		Positive	Negative	Positive	Negative
Truth	Positive	46	5	46	5
	Negative	20	185	27	178

		Contains environmental genomes		Missing environmental genomes	
Only genomes from literature review		Precited		Predicted	
		Positive	Negative	Positive	Negative
Truth	Positive	42	9	42	9
	Negative	20	185	27	178

Figure S3. Confusion matrices illustrating SourceApp performance with and without environmental genomes from aquatic prokaryotes. SourceApp was run with the same parameter set selected through parameter tuning for source attribution. The different databases indicated are described in detail in Table S1. The increased false negative frequency in the bottom panel of confusion matrices is due in part to the absence of a septage source category while the increase in false positives on the right hand side is due, primarily, to false wastewater calls.

Predicted portions of synthetic community spiked into freshwater; **without** environmental screen

Genome	3% spike-in	23% spike-in	75% spike-in	Expected
Lactobacillus fermentum	21.8%	20.1%	21.4%	21.6%
Staphylococcus aureus	21.3%	18.2%	19.9%	15.2%
Listeria monocytogenes	18.6%	16.8%	18.5%	13.9%
Enterococcus faecalis	9.1%	11.0%	13.9%	14.6%
Bacillus subtilis	9.7%	9.5%	9.2%	10.3%
Salmonella enterica	7.4%	8.6%	6.5%	8.7%
Escherichia coli	8.6%	11.3%	7.3%	8.5%
Pseudomonas aeruginosa	3.5%	4.3%	3.0%	6.1%
*Saccharomyces cerevisiae**	0.0%	0.2%	0.4%	0.6%
*Cryptococcus neoformans**	0.0%	0.0%	0.2%	0.4%

Predicted portions of synthetic community spiked into freshwater; **with** environmental screen

Genome	3% spike-in	23% spike-in	75% spike-in	Expected
Lactobacillus fermentum	22.6%	21.0%	22.0%	21.6%
Staphylococcus aureus	22.1%	19.0%	20.4%	15.2%
Listeria monocytogenes	19.3%	17.5%	19.0%	13.9%
Enterococcus faecalis	9.4%	11.5%	14.3%	14.6%
Bacillus subtilis	10.0%	9.9%	9.4%	10.3%
Salmonella enterica	7.7%	8.9%	6.6%	8.7%
Escherichia coli	5.2%	7.4%	4.6%	8.5%
Pseudomonas aeruginosa	3.6%	4.5%	3.1%	6.1%
*Saccharomyces cerevisiae**	0.0%	0.2%	0.4%	0.6%
*Cryptococcus neoformans**	0.0%	0.0%	0.2%	0.4%

Figure S4. Results from analyzing metagenomes obtained from the three synthetic community (Zymo) spike-in mesocosms with SourceApp against the databases built without (top) and with (bottom) an environmental genome set (for genome database information see Table S2). Note that *Escherichia coli* was the only species in the mock community to be reported as cross-reactive with another species in the environmental genome set according to "sourceapp build" during database construction. Further information on mesocosm composition and genome databases can be found in Tables S1 and S2, respectively. * - indicates Eukaryotic species.

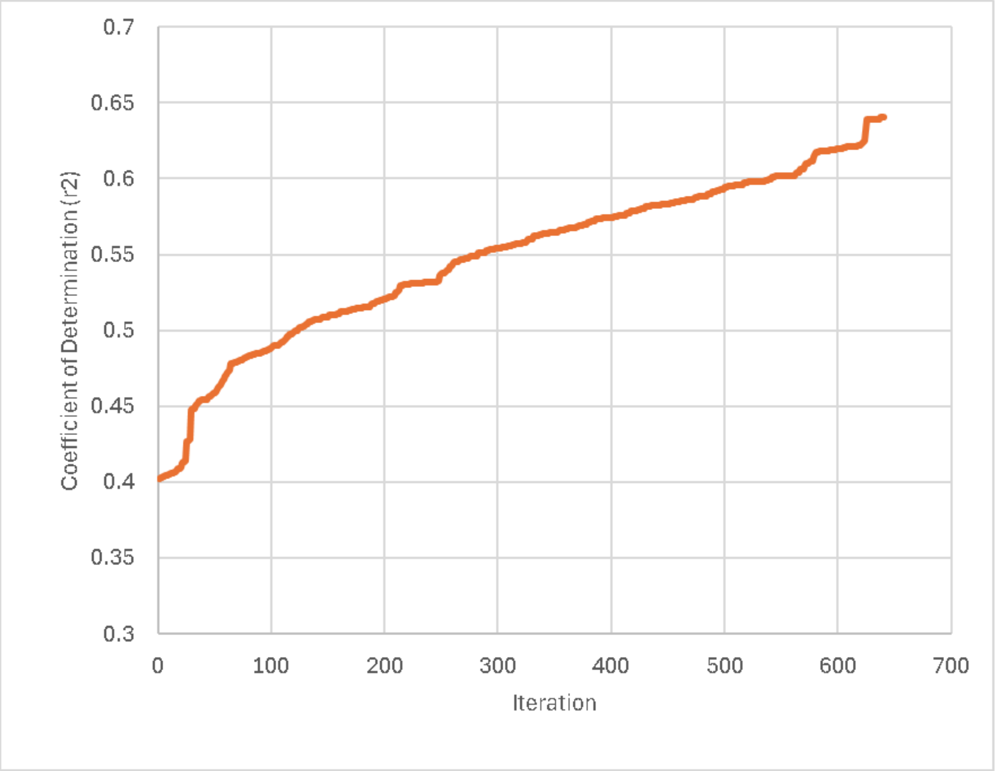

Figure S5. Parameter tuning results for use of SourceApp for cell fractionation. See also Table S4. The coefficient of determination (r^2) reported for each iteration was the result of linearizing predicted cell fractions to ground truth cell fractions. These results show that across the 640 SourceApp parameter tuning iterations there was a set of parameters which produced cell fraction estimations which were easiest to correct by multiplication of a single scalar (slope).

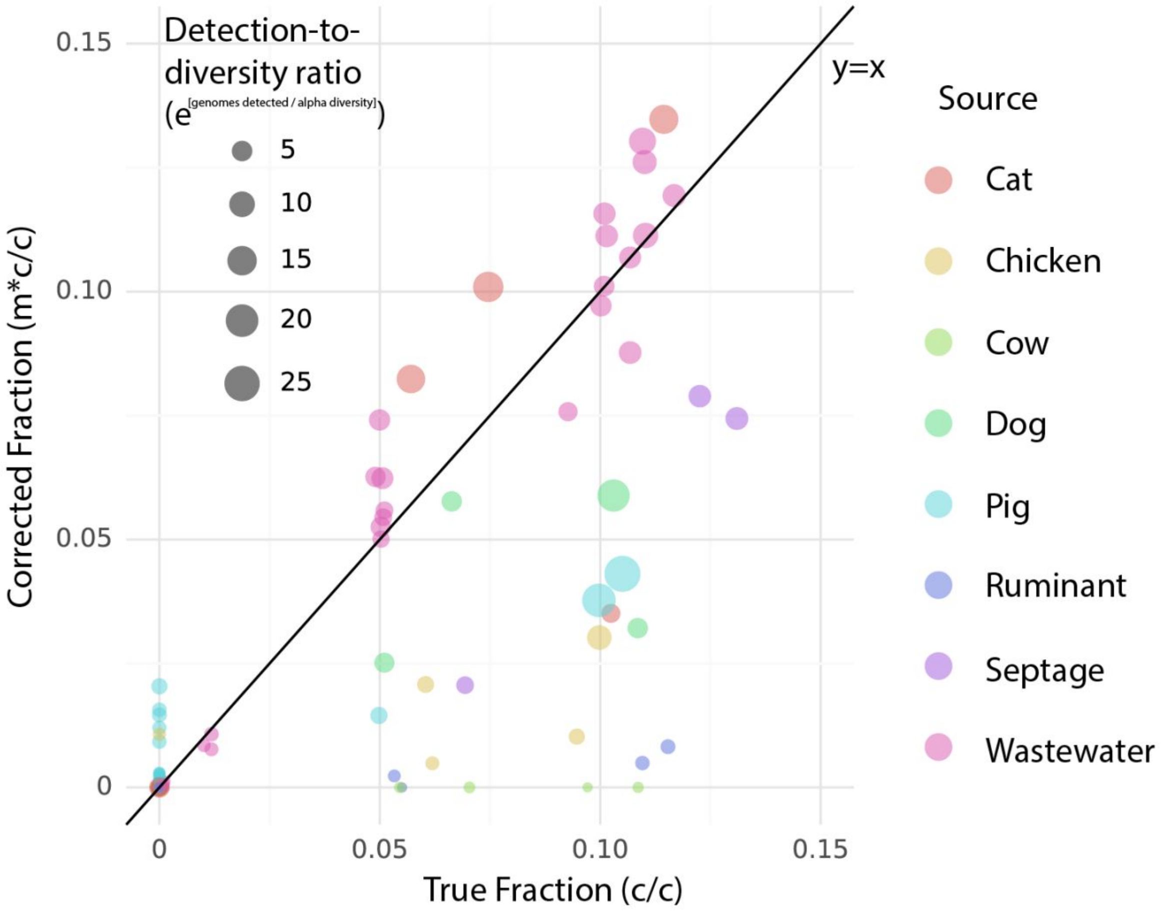

Figure S6. Fecal cell fractions across all sources and all mesocosms after correction with the slope (m) of the linear model producing least residuals found via parameter tuning. See also Figure S5 and Table S4.

Synthetic community spike-in cell fractions

	3% spike-in	23% spike-in	75% spike-in
Predicted:	1%	7%	28%
Expected:	3%	23%	75%

Figure S7. Total cell fraction predicted end expected values for the three synthetic spike-in mesocosms. Cell fraction values were found with equation 3 using the total cell density provided by the manufacturer (~1.4x10^{10} cells/mL) and direct counting of the lake water used in mesocosm construction.

Fluorescence Microscopy

All microscopy was completed within 24 hours of sampling and these efforts aimed to produce approximate estimates for original cell densities in the fecal samples and environmental matrix (i.e., lake water) to guide subsequent mesocosm construction. Each of the fecal samples were serially diluted from 10^{-1} to 10^{-4} (v/v). Approximately 0.5mL of DAPI solution (1 mg/mL) was added to all dilutions and incubated in the dark for 15 minutes, vortexing before and after incubation. After staining, the sample was mixed by pipette with 1 mL sterile PBS before vacuum filtering through 25mm 0.2 μm black polycarbonate membrane (Isopore, Millipore) with two subsequent sterile PBS washes to encourage formation of even and confluent layers of cells on the filter membrane surface. Lake water was processed as above but without dilution and using 1.5mL total to ensure adequate cellular coverage of the filter for counting purposes (i.e., to avoid fields without cells). For each sample, dilutions which were too numerous to count were discarded, and subsequent dilutions examined until countable. When an appropriate dilution for counting was found, twenty images were randomly taken across the surface of the filter using a fixed field of view (89.5 μm by 67.1 μm) in the Zeiss Zen software suite using an equipped AxioCam MRm. Each set of images was counted by four different individual researchers with results recorded and averaged to produce an average number of cells per image. Using this average, the total number of cells filtered was calculated by extrapolating the image's field-of-view across the entire inner diameter formed on the filter by the filtration apparatus (assuming an even distribution of cells thereon; 1.6 cm inner diameter). This value for the total number of cells captured by filtration was then used, considering any dilutions, to back calculate the original sample's cell density (in cells/mL).

DNA extraction and sequencing

Filter membranes containing mesocosm biomass were bead beaten prior to extractions using an MP Biomedicals FastPrep-24 instrument with three rounds of beating for 60 seconds and 60 seconds of ice incubation in between rounds. Extracts were eluted in 100 µL of Buffer CD6 and stored at -80°C for approximately three months prior to library preparation and sequencing. Fecal slurries used as sources in the mesocosm experiments were thawed at 4°C for less than 24 hours prior to DNA extractions. Samples were then extracted using the High molecular weight (HMW) DNA was eluted in 50 µL of elution buffer and replicates from the same fecal source were combined to increase yield for sequencing. Pooled HMW DNA was then ethanol precipitated to concentrate and purify DNA prior to library preparation. DNA pellets were resuspended in 75 µL of Zymo elution buffer, then analyzed for quality using a NanoDrop spectrophotometer, Qubit 1x HS dsDNA quantification kit, and gel electrophoresis with the NEB 1 kb Extend ladder. Extracts were stored at -20°C prior to library preparation and sequencing on the Oxford Nanopore MinION. HMW DNA purified extracts were thawed on ice on the day of library preparation and sequencing. Some extracts were purified and/or concentrated using a Blue Pippin or Vacufuge prior to library preparation to achieve the required DNA input. Single extracts were prepared using the ONT Rapid Sequencing kit (SQK-RAD114), sequenced on Flongles (ONT #FLO-FLG114, r10), and subsequent basecalling performed with dorado. All data from replicate runs of a given fecal slurry extract were combined during bioinformatic analysis.

The mesocosm samples containing fecal spike-ins and synthetic community spike-ins were sequenced in the same manner as the previously reported short read metagenomes from the fecal slurries[1]. That is, all Illumina sequencing libraries were prepared from 50 ng of input DNA for

each sample using the Illumina DNA prep kit with unique dual indexing as recommended by the manufacturer. Library concentrations were determined using the Qubit 1X dsDNA High Sensitivity kit (ThermoFisher Scientific) and Qubit 2.0 fluorometer and average insert size was determined by sizing using the Agilent 2100 Bioanalyzer and a High Sensitivity DNA Analysis kit. Libraries were pooled in an equimolar mixture and sequenced by the Georgia Institute of Technology Molecular Evolution Core on an Illumina NovaSeq 6000 instrument for 2 × 150-bp paired-end reads. Adapter trimming and demultiplexing were carried out on the instrument.

Parameter tuning and scoring

To improve performance, we systematically evaluated the same set of parameters (Tables S3 and S5) across attribution and fractioning separately. The minimum percent identity and read alignment fraction necessary to accept a mapping were evaluated from 85 to 99% and 30 to 90%, respectively. Masking reference genomes has become a common practice in ecological surveys of environmental microbiomes where there is some degree of uncertainty regarding how well a reference genome matches the population *in situ*[2]. Thus, we tested various degrees of genome masking, from 0 to 20%. Additionally, as part of parameter tuning, we also examined several choices for a minimum cell fraction cutoff for attributing a particular source, ranging from 0% (no cutoff) to 0.1% (see 2.7 above). Selections for intervals within these parameters were balanced with the number of necessary iterations to examine a parameter's effect. The final number of parameter combinations, and therefore iterations, was 640 across the four parameters: minimum read-to-genome alignment identity, minimum alignment-to-read overlap, reference genome masking, and minimum relative abundance cutoff.

For source attribution, optimal parameter set was then selected by scoring results and selecting those parameters resulting in the greatest negative predictive value (NPV), i.e., the parameters which yielded highest precision within the range of greatest specificity. For cell fractioning, linear models were constructed from estimated cell fractions using each parameter combination and then fit to the ground truth cell fractions. The coefficient of determination (r^2) of the resulting predictions made by each linear model for each parameter set was measured and the process repeated until all parameter combinations were examined in this way. The best performing parameter set was selected based on producing data with the least residuals after linear regression and thus reported the largest coefficient of determination.

As mentioned, classification metrics such as negative predictive value, sensitivity, and specificity were used to examine the success of source attribution for SourceApp and other tools (discussed below). We produced values for these terms via counts of positive or negative source attribution compared to the ground truth according to the following:

$$\text{Sensitivity} = \text{True Positives} / (\text{True Positives} + \text{False Negatives})$$

$$\text{Specificity} = \text{True Negatives} / (\text{True Negatives} + \text{False Positives})$$

$$\text{Negative Predictive Value} = \text{True Negative} / (\text{True Negative} + \text{False Negatives})$$

Tool-to-tool Comparisons

We compared SourceApp's source attribution and apportionment capabilities to those of metaSourceTracker, FEAST, and decOM. Taxonomic profiles for all short read metagenomes were generated with Kraken2 against its "standard" database. The results for each mesocosm and fecal slurry short-read metagenome were concatenated into a single tabular file according to

taxonomic identifiers (rows) and samples (columns). This data is stored as Table S6 within Supporting Information. Metagenomic data was processed via kmtricks as described in the decOM documentation to prepare *k*-mer tables for each each source and sink. Taxonomic profiles (for metaSourceTracker and FEAST) and *k*-mer count tables (for decOM) were then provided to each tool along with necessary mapping/metadata files with information on the source/sink identity of each sample recorded in Supporting Information (Table S7). For all tools, the negative control mesocosm was flagged as an additional "source" representing the environmental matrix (i.e., lake water). FEAST and decOM were operated with default parameters. metaSourceTracker was called with "sourcetracker 2 gibbs" and the "—diagnostics –draws_per_restart 2" flags. FEAST and metaSourceTracker were run in triplicate using the same inputs across each replicate.

The resulting proportions were analyzed, dropping the environmental portion, and re-normalizing to compare fecal portions between all tools. Resulting proportions below 0.01% for a source were dropped. For measuring source attribution, sensitivity and specificity were scored based on the presence or absence of each source across all sinks compared to the ground truth. For assessing source apportioning, the Spearman rank correlation coefficient was calculated between each tool's results and the ground truth portions. These same methods were also applied to the results of SourceApp after its development, parameter tuning, and database construction. The raw results of each tool are recorded in the Supporting Information (Table S7) as well as the resulting source attribution and apportioning scores (Table S8).

SourceApp Development and Database Construction

The two main functions of SourceApp, as well as expected inputs and outputs, are summarized in Figure 1 and briefly described below. The first function includes a fully automated and parameterized metagenomic workflow for source attribution, apportionment, and cell fractioning. This pipeline requires a properly formatted genomic database. Such a database can be constructed by the user via the second function of SourceApp and requires input of prokaryotic genomes (draft or complete) with known source associations as a text file. In this way, SourceApp's database construction function allows users to supply their own genomes, rejecting the default database, or supplementing it with genomes from their own work. Genomes provided by the user can be associated with sources already present in the database or part of new, user-defined source categories which will be created and curated by SourceApp on-the-fly. The primary database generated herein serves users as the default database and can be used without providing any additional genomes (discussed below).

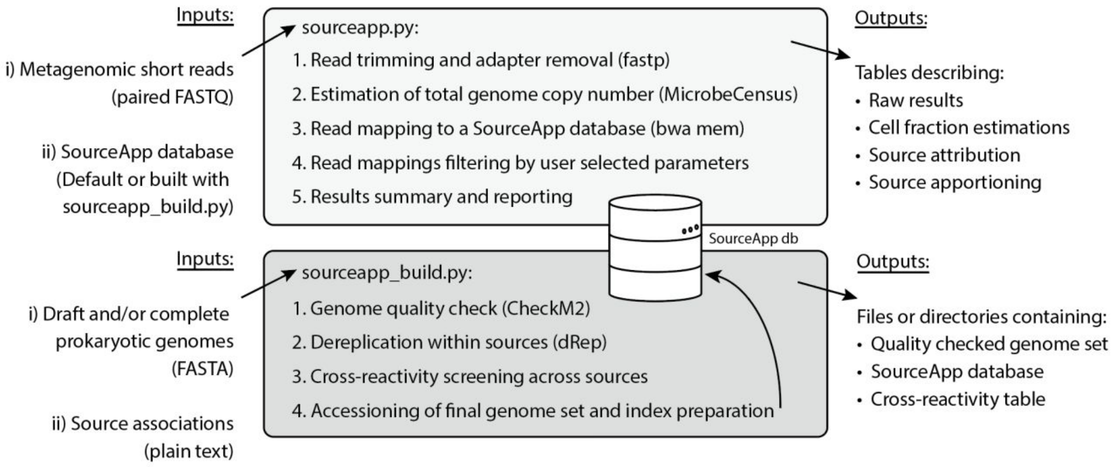

Figure S8. Workflows associated with SourceApp's two main subroutines. Not all available options are shown, and some subroutines can be disabled.

Database construction is a vital component of this pipeline and is where source specificity and cross reactivity of genomes are determined. By default, cross reactivity is based on clustering genomes across sources at the ≥95% ANI level. Genomes within cross-reactive clusters are not removed but simply flagged as “cross-reactive” to assist with downstream analysis while singletons are flagged as “source-specific”. To illustrate the role of the genome database in our metagenomic FST approach, we constructed multiple genome databases for use by SourceApp. By using multiple genome databases, the impact of 1) site specific genomes (e.g., MAGs recovered by sequencing spike-in slurries) and 2) environmental genome sets could be ascertained. The various databases constructed as part of these efforts are summarized in supporting information and the content presented below focuses on use of the best performing database while results for other databases can be found in the supporting information (Figure S2).

The logic employed by SourceApp for use in attribution, apportioning, or fractioning are described in methods and materials section 2.6.

SI Citations

(1) Lindner, B. G.; Choudhury, R. A.; Pinamang, P.; Bingham, L.; D’Amico, I.; Hatt, J. K.; Konstantinidis, K. T.; Graham, K. E. Advancing Source Tracking: Systematic Review and Source-Specific Genome Database Curation of Fecally Shed Prokaryotes. *Environ. Sci. Technol. Lett.* **2024**, *11* (9), 931–939. https://doi.org/10.1021/acs.estlett.4c00233.

(2) Meziti, A.; Rodriguez-R, L. M.; Hatt, J. K.; Peña-Gonzalez, A.; Levy, K.; Konstantinidis, K. T. The Reliability of Metagenome-Assembled Genomes (MAGs) in Representing Natural Populations: Insights from Comparing MAGs against Isolate Genomes Derived from the Same Fecal Sample. *Appl. Environ. Microbiol.* **2021**, *87* (6), e02593-20. https://doi.org/10.1128/AEM.02593-20.
